# Sexual orientation, gender identity and cardiometabolic risk: a narrative review

**DOI:** 10.1007/s00125-025-06572-7

**Published:** 2025-10-21

**Authors:** Meredith S. Duncan, Lauren B. Beach, Hill L. Wolfe, Arjee Restar, Adovich S. Rivera, Carl G. Streed

**Affiliations:** 1 Department of Biostatistics, University of Kentucky, Lexington, KY USA; 2 Department of Medical Social Sciences, Northwestern University Feinberg School of Medicine, Chicago, IL USA; 3 Department of Biomedical Informatics & Data Science, Yale School of Medicine, New Haven, CT USA; 4 Weitzman Institute, Moses Weitzman Health System, Washington, DC USA; 5 Department of Social and Behavioral Sciences, Yale University, New Haven, CT USA; 6 Section of General Internal Medicine, Boston University Chobanian and Avedisian School of Medicine, Boston, MA USA; 7 GenderCare Center, Boston Medical Center, Boston, MA USA

**Keywords:** Cardiometabolic, Cardiovascular, Diabetes, Gender identity, Hormones, Review, Sexual orientation, Stress

## Abstract

Recent reports estimate that approximately 9% of adults globally identify as a sexual and gender minority (SGM), including lesbian, gay, bisexual, transgender or queer (LGBTQ+) people. While research has shown that SGM populations experience health disparities, few studies have focused on cardiometabolic risk and outcomes, particularly with regard to diabetes and its sequelae. Using narrative review methods, we describe the epidemiology of cardiometabolic risk factors and outcomes in SGM populations, with a focus on literature resulting from meta-analyses and analyses of large samples published between 2017 and 2025. We also propose recommendations for follow-up and monitoring of metabolic and cardiovascular risk in SGM populations. Available data show varying risk for cardiometabolic conditions across SGM subpopulations. Studies of UK data have noted that sexual minority women have an increased risk of diabetes compared with heterosexual women, while data from the USA reveal ambiguous results depending on the data source. Transgender adults were found to be at increased risk for diabetes compared with cisgender comparators in UK data sources but not in US sources. For chronic kidney disease (CKD), risk varied by subpopulation and data source. Notably, SGM subpopulations in the UK were reported to have an increased risk of CKD while similar populations in the USA appear to have no difference in risk compared with cisgender, heterosexual comparators. Similarly, risk for CVD among sexual minority populations varied by data source. However, both US and UK data suggest that transgender adults are at increased risk of CVD. Subsequent research is needed to further characterise these disparities and longitudinal cohort studies must be explicitly inclusive of SGM populations. Ideally, SGM-specific longitudinal cohort studies should be executed to more accurately and effectively characterise cardiometabolic risk, outcomes and potential interventions in these populations.

**Graphical Abstract:**

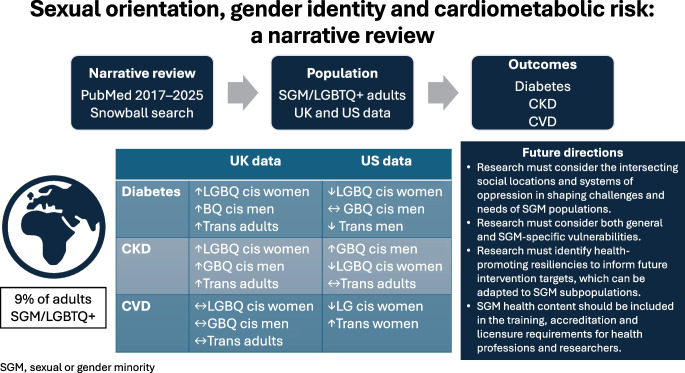

**Supplementary Information:**

The online version contains peer-reviewed but unedited supplementary material including a slide of the figure for download, available at 10.1007/s00125-025-06572-7.

## Introduction

Sexual and gender minority (SGM) populations account for 9% of adults globally (including lesbian, gay, bisexual, transgender or queer [LGBTQ+] people) [1]; this proportion is expected to grow as younger generations are more likely to identify as SGM than older people [1, 2]. Studies show that SGM populations experience health disparities [3], but few studies have focused on cardiometabolic conditions [4–6]. Previous reviews of cardiometabolic health in SGM individuals describe conflicting results and suggest that, while SGM populations experience heightened burdens of cardiometabolic risk factors that vary across sexual orientations and gender identities, their incidence of cardiometabolic outcomes may not significantly differ from that in the population at large [4, 5, 7].

The current narrative review describes the epidemiology of cardiometabolic risk factors and outcomes in SGM populations in the UK and USA, with a focus on literature resulting from meta-analyses and analyses of large data sources (e.g. national surveillance systems) published between 2017 and 2025. In addition, we examine how limitations in study design may explain mixed findings regarding prevalence of diabetes, comorbidities and complications. The text box provides a glossary of terminology to facilitate a shared set of definitions related to the study of sexual orientation and gender identity.


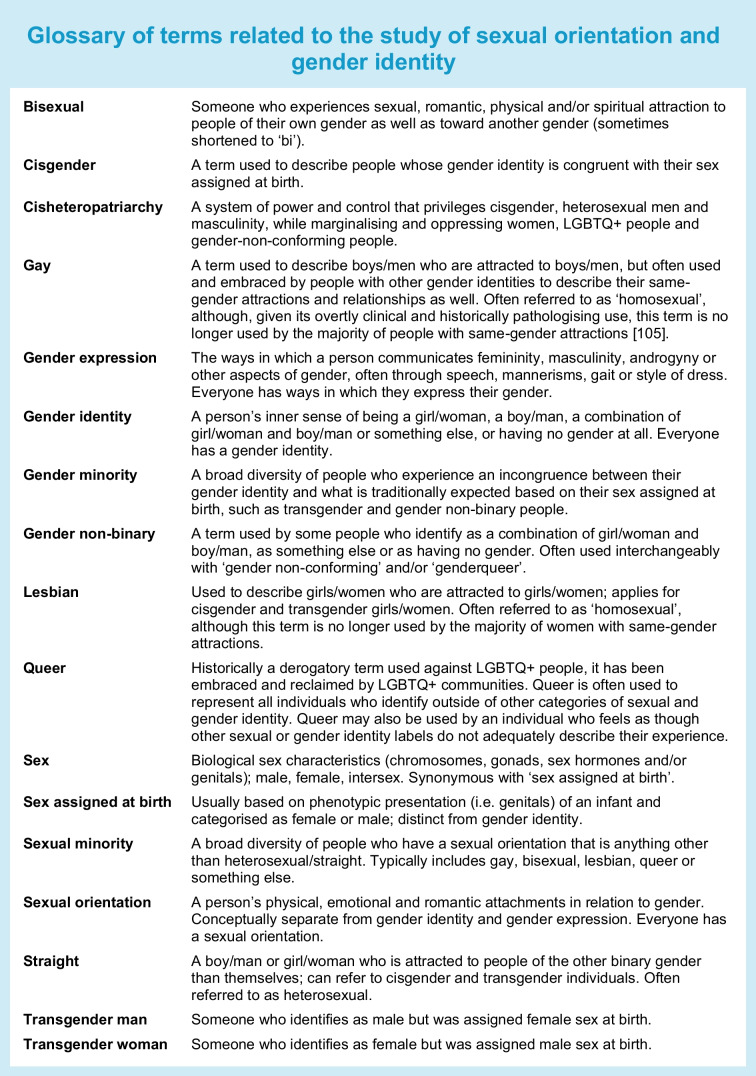


## Methods

### Search strategy and included literature

We performed separate literature searches for the various topics covered in this review; search strategies are provided in ESM Table 1. To keep the review focused and contemporary, we used PubMed as our primary database and prioritised publications from 2017 to 2025 with large sample sizes or meta-analyses to reduce the bias that may arise when reporting results from single studies with small sample sizes. We limited our review to publications reporting results from studies conducted in the UK and USA. To compensate for using PubMed as our primary database, we also reviewed the references cited in identified publications and included those that were relevant. As this is a broad narrative review covering several aspects of cardiometabolic health in SGM individuals, rather than a systematic review, there were no rigorous inclusion/exclusion criteria. Thus, other publications could have been included; the publications included are frequently cited and were included to highlight the variation that exists in the current literature on these topics.

### Conceptual model

**Fig. 1:**
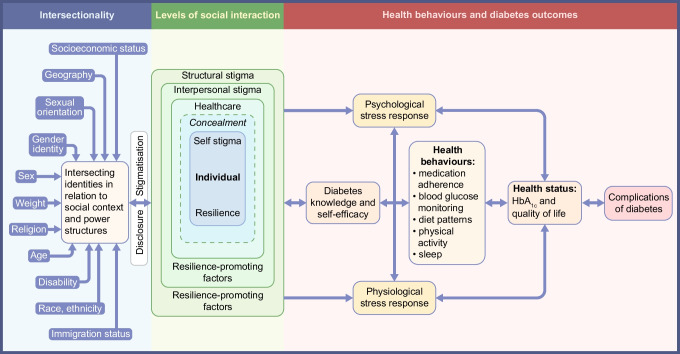
Conceptual model of intersectional stigma and multilevel minority stress and cardiometabolic risk factors and outcomes in SGM populations. In brief, the conceptual model depicts intersectional stigmatisation operating dynamically in relation to resilience-promoting factors at structural, interpersonal and individual levels (nested boxes) that influence distal and proximal minority stressors. When stigmatisation rather than resilience is the dominant effect within the model (as generally posited for SGM populations), the resulting net effect of intersectional stigmatisation contributes to poor SGM population health and health disparities via minority stress-linked pathways of psychological distress and cardiometabolic behavioural risk factors. These pathways contribute to clinical cardiometabolic risk factors and outcomes. They also add to the higher chronic stress burden associated with the development of clinical cardiometabolic risk factors and disparities, including diabetes and complications onset among SGM populations with intersecting marginalised identities. When resilience is the dominant effect, the negative health effects of these minority stress pathways are decreased, resulting in the lessening of diabetes risk factors, incidence and complications. This figure is available as a downloadable slide

Foregrounding the Black feminist frame of intersectionality [8], our conceptual model (Fig. 1) depicts how intersectional stigma and multilevel minority stress are connected to disparities in cardiometabolic risk factors and outcomes in SGM populations [4, 5, 9]. Briefly, one’s multiple dimensions of positionality (e.g. race and ethnicity, sex, gender, sexual orientation, disability) are subjected to societal systems of privilege, power and oppression (e.g. cisheteropatriarchy, structural racism, ableism, homophobia, transphobia), producing intersectional stigma. The net effects of intersectional stigma are manifested across socioecological levels and influence health behaviours, chronic stress and inflammation, which shape blood glucose levels, HbA_1c_, quality of life, and diabetes incidence, prevalence, comorbidities and complications. The model applies at both individual and population levels. When applying the model in public health, expected outcomes are predicted to vary, with groups facing increasing levels of intersectional stigma (e.g. Black bisexual women) hypothesised to have worse health. Notably, structural, social and internalised stigma can be modified by resilience-promoting factors at multiple levels. Cardiometabolic health outcomes of a given individual or group should be viewed as the net effect of modifiable structural, social and individual processes that can be disrupted or supported to promote health equity. Despite the paucity of research regarding resilience and diabetes, related research in cardiovascular health has noted potential associations. As such, we have elected to ensure that resilience is incorporated in the conceptual model to encourage future study of this component of well-being.

Notably, intersectionality is a necessary component of the conceptual model, as risk factors and outcomes, as well as social determinants of health and other key sociodemographic factors, vary by racial and ethnic groups within SGM populations. Therefore, we report using our ‘primary lens’ of SGM status but must highlight that we cannot capture the heterogeneity of findings in these populations without also addressing these additional intersectional factors; SGM health is directly tied to the full, holistic social position of SGM people within a given social context.

## Results

### Risk factors for diabetes

Modifiable risk factors for diabetes, including adiposity, poor-quality diet and lack of physical activity, are well established [10]. Studies also report associations between substance use, deficient sleep and HIV infection and diabetes incidence in the general population [10]. Consistent with our conceptual model, studies have shown that multilevel minority stress in SGM adults is associated with all of the aforementioned diabetes risk factors [11]. However, much of the literature assessing the association of sexual orientation and/or gender identity with diabetes risk factors focuses on this single dimension of positionality. Therefore, we report intersectional data when they exist but primarily focus on the distribution of diabetes risk factors in SGM populations compared with heterosexual and/or cisgender individuals overall. A summary of the following information on risk factors is displayed in Table 1.

**Table 1:** Diabetes risk factors in SGM populations

Risk factor	Country	First author (year)	Data source	Finding
BMI	UK	Semlyen (2020) [13]	Meta-analysis of 12 studies	Lesbian and bisexual women at higher risk of obesity or overweight than heterosexual women
				Gay men at lower risk of obesity or overweight than heterosexual men
		Dinos (2021) [15]	2021 Health Survey for England	51% of sexual minority adults overweight or obese vs 63% of heterosexual adults
	USA	Gonzales (2017) [14]	2014–2015 BRFSS	Lesbian and bisexual women more likely to have obesity than heterosexual women
Diet	UK	Calzo (2018) [19]	5048 adolescents in the Avon Longitudinal Study of Parents and Children	‘Mostly heterosexual’ boys and girls had greater risk of dieting, overeating and binge eating than ‘completely heterosexual’ adolescents of the same sex
				Gay and bisexual boys had increased risk of dieting vs heterosexual boys
				Lesbian and bisexual girls had increased risk of binge eating vs heterosexual girls
	USA	Diemer (2015) [20]	American College Health Association–National College Health Assessment II	Transgender students had greater odds of past-year eating disorders and past-month use of diet pills or laxatives and vomiting vs heterosexual women
				Sexual minority men had greater odds of past-year eating disorders vs heterosexual men and women
				Sexual minority men at lower odds of past-month diet pill or laxative use and vomiting vs heterosexual women
		Nagata (2020) [103]	PRIDE study	Among genderqueer/non-binary individuals and transgender men, lifetime use of appearance- and performance-enhancing drugs was associated with dietary restraint and binge eating
		Gibb (2021) [22]	2003–2016 NHANES	Moderate and severe food insecurity significantly higher among sexual minority individuals than heterosexual peers
Physical activity	UK	Sport England (2024) [23]	Active Lives Survey	Greater proportion of sexual minority adults complete 150+ min of moderate to vigorous physical activity per week than heterosexual adults
		Jones (2018) [27]	360 transgender and 314 cisgender adults	TGD individuals engaged in less physical activity than cisgender individuals
				Among TGD individuals, those on GAHT were more engaged in physical activity than those not on GAHT
	UK/USA	Herrick (2018) [25]	Systematic review of 35 studies	Sexual minority men generally possess greater physical activity levels than heterosexual men, often motivated by a perceived body ideal of being thin and/or muscular
				Sexual minority women typically have decreased physical activity levels vs heterosexual women, predicated on a social norm that emphasises bodily acceptance
	USA	Fricke (2019) [24]	Kaiser Permanente Northern California Member Health Surveys	Sexual minority women engage in more frequent physical activity and at higher intensity levels than heterosexual women
				Sexual minority men engage in more frequent but less intense physical activity than heterosexual men
		Rosendale (2024) [26]	2007–2016 NHANES	No difference in physical activity levels among sexual minority adults vs sex-matched heterosexual counterparts
Substance use	UK	Kneale (2020) [32]	Meta-analysis of 23 studies	Sexual minority adults have greater prevalence of smoking and high-frequency alcohol consumption than heterosexual adults
		Meads (2023) [31]	Systematic review of 20 studies	Harmful alcohol use is more prevalent in SGM people than heterosexual people
	UK/USA	Ruppert (2021) [29]	Scoping review of 55 studies	Tobacco use is higher among TGD populations than cisgender individuals
				Transgender men may exhibit heavier episodic drinking than cisgender populations
				Transgender men may also have higher risk of alcohol use disorders than transgender women
	USA	Taylor (2022) [33]	2013–2018 NHIS	Lesbian and bisexual women are more likely to smoke and binge drink than heterosexual women
Sleep	UK	Wong (2024) [36]	Millennium Cohort Study	SGM adolescents have poorer sleep patterns than heterosexual cisgender peers
	UK/USA	Butler (2020) [35]	Narrative review of 31 studies^a^	Sexual minority adults have more sleep disturbances and a lower quality of sleep than heterosexual adults
				Unclear whether gay men have better or worse sleep than heterosexual men
	USA	Dai (2019) [34]	2014 BRFSS	Gender non-conforming individuals sleep less than individuals of other gender identities
		Almazan (2019) [37]	2013–2017 NHIS	Sexual minority adults had more difficulty staying asleep than heterosexual adults

^a^Additionally includes studies from France (*n*=2) and Germany (*n*=1)

BRFSS, Behavioural Risk Factor Surveillance System; PRIDE, Population Research in Identity and Disparities for Equality

#### BMI

Elevated BMI is the most common risk factor for type 2 diabetes; 90% of adults in the UK and USA with diabetes have a BMI ≥25 kg/m^2^ [12]. A meta-analysis of 12 studies from the UK reported that both lesbian (OR [95% CI] 1.41 [1.16, 1.72]) and bisexual (OR [95% CI] 1.24 [1.03, 1.48]) women were more likely to have overweight or obesity than heterosexual women, while gay men had a lower risk than heterosexual men (OR [95% CI] 0.72 [0.61, 0.85]) [13]. In the USA, Behavioral Risk Factor Surveillance System (BRFSS) data support the findings of this UK meta-analysis [14]. In contrast, the 2021 Health Survey for England found that a lower proportion of sexual minority adults were overweight or obese (51%) than heterosexual adults (63%) [15]. Additional data from the USA suggest that BMI in SGM populations may vary by race and ethnicity. Wave IV of the US National Longitudinal Study of Adolescent Health (Add Health) found no sexual orientation differences in BMI among Black/African American women, while White and Latina bisexual women were found to have a higher BMI than same race and ethnicity heterosexual individuals [16]. However, in 2002–2016 National Health and Nutrition Examination Survey (NHANES) data, Black and White lesbian women and bisexual women of all races/ethnicities had a higher BMI than White heterosexual women [17]. Among transgender and gender-diverse (TGD) individuals, gender-affirming hormone therapy (GAHT) may be linked to changes in obesity prevalence [4].

#### Diet

While SGM individuals and their heterosexual cisgender peers have similar dietary behaviours, some SGM subgroups demonstrate differing dietary behaviours, patterns and quality as well as risk for disordered eating [18]. A study of UK adolescents reported that ‘mostly heterosexual’ boys and girls had approximately twice the risk of dieting, overeating and binge eating compared with ‘completely heterosexual’ adolescents of the same sex; that gay and bisexual boys had 2.67 times the odds of dieting compared with heterosexual boys; and that lesbian and bisexual girls had 4.43 times the odds of binge eating compared with heterosexual girls [19]. Similar results were observed in a study of US college students; this study also reported that transgender students had 4.62 times the odds of a past-year eating disorder diagnosis compared with heterosexual cisgender female students [20]. The US-based Population Research in Identity and Disparities for Equality (PRIDE) study reported that, among genderqueer/non-binary individuals and transgender men, lifetime use of appearance- and performance-enhancing drugs was associated with dietary restraint and binge eating [21]. Further, 2003–2016 NHANES data demonstrate that moderate and severe food insecurity is 20–70% higher among sexual minority individuals compared with heterosexual peers [22]; these findings persisted after adjusting for multiple sociodemographic factors and are likely to be driven by higher rates of poverty and homelessness experienced in sexual minority compared with heterosexual adults [22].

#### Physical activity

Physical activity varies across SGM subpopulations [18]. The UK Active Lives Survey 2022–2023 found that 72% of gay/lesbian and 70% of bisexual adults completed 150+ min of moderate to vigorous physical activity per week compared with 64% of heterosexual adults [23]. Data on insured residents in Northern California revealed that sexual minority women engaged in more frequent physical activity at higher levels of intensity, and for an average of 13 min more per week, than heterosexual women, but that, although sexual minority men also engaged in physical activity more frequently than heterosexual men, the intensity level was lower [24]. In contrast, a systematic scoping review demonstrated that sexual minority women may have lower physical activity levels than heterosexual women due to homophobia and/or wider acceptance of a variety of body sizes (e.g. some butch women embracing a larger size) [25]. Further, while some sexual minority men had increased physical activity levels, to cultivate an ‘ideal’ athletic body, other image classifications among gay men, such as ‘femme’, may suggest lower physical activity levels [25]. An NHANES analysis revealed no difference in physical activity levels among sexual minority adults compared with sex-matched heterosexual counterparts; these findings remained consistent across race and ethnicity [26]. Some evidence suggests that TGD individuals may be less active than their cisgender counterparts [27], possibly due to an increased risk of victimisation based on gender identity within team sport settings. However, a systematic review focused on TGD populations revealed that, despite the gendered nature of several team sports and sport facilities, engaging in individual physical activities can promote further exercise when spaces are perceived as comfortable and accessible [28]. Finally, although not every TGD person desires or accesses transition-related health services, TGD individuals may participate in physical activities to achieve surgical candidacy [28] or as a result of a positive body image from GAHT [27].

#### Substance use: alcohol, tobacco and other drugs

Numerous studies demonstrate that SGM individuals, as a group, are at increased risk of dependence on tobacco, alcohol and other drugs compared with their non-SGM counterparts, but this risk may differ by sex, sexual orientation and gender identity [29–32]. A meta-analysis of 23 studies in the UK reported that lesbian, gay and bisexual (LGB) adults had 1.26 and 1.21 times the odds of smoking and high-frequency alcohol consumption, respectively, in unadjusted models compared with heterosexual adults [32]. However, in sex-specific analyses, only LGB women had a greater odds of smoking compared with heterosexual female participants (OR [95% CI] 1.23 [1.05, 1.44]); there was no significant association between sexual orientation and substance use in men nor between sexual orientation and alcohol consumption in women. Data from the US National Health Interview Survey (NHIS) suggest that lesbian and bisexual women are more likely to smoke cigarettes and binge drink than heterosexual women, but these results differed by partnership status [33]. Regarding TGD populations, tobacco use is higher among TGD populations than cisgender individuals and transgender men may exhibit heavier episodic drinking than cisgender populations [29]. Multiple studies show that transgender men may also have a higher risk of alcohol use disorders than transgender women [29]. Illicit drug use and prescription drug misuse is also higher among TGD individuals than the general US population [29]. Most research on illicit drug use among TGD populations has focused on transgender women, showing elevated use of several drugs, especially methamphetamine, compared with transgender men [29].

#### Sleep

Poor sleep quality is associated with several adverse health risks [34] and findings on sleep health and related behaviours in SGM subpopulations are mixed [35]. The majority of studies reveal that sexual minority adults have more sleep disturbances and a lower quality of sleep than heterosexual adults [35]. In a UK study of adolescents, both gender minority and sexual minority adolescents had worse sleep patterns than their heterosexual cisgender peers, with gender minority adolescents displaying the poorest sleep patterns [36]. Consistent results were observed in an analysis of 2013–2017 US NHIS data, which found that sexual minority individuals across all racial and ethnic categories had more trouble staying asleep than heterosexual peers [37]. However, some studies reveal higher sleep quality in gay men than heterosexual men, while other studies demonstrate shorter periods of sleep and greater sleep disturbances for gay men [35]. A narrative review highlighting limitations among sleep research focused on SGM individuals reported the use of overlapping secondary datasets, centring research on sleep quality as opposed to other important sleep factors (e.g. sleep apnoea, hypersomnia), and few studies including gender minority individuals, rather than just sexual minority individuals [35]. Despite the paucity of literature, poor sleep quality has been a consistent finding among transgender women and men [35, 38]. Further, gender non-conforming individuals have reportedly shorter sleep periods (≤5 h/day) than other cisgender and transgender subgroups [34].

#### HIV

With antiretroviral therapy (ART), people living with HIV (PLWH) are ageing well but also at risk of developing chronic conditions, including diabetes. HIV and exposure to ART regimens may place PLWH at higher risk for diabetes; the association between ART and diabetes incidence may be mediated through ART-induced weight gain [39, 40]. HIV disparities impact a variety of SGM subpopulations and, as such, diabetes risk may be concomitantly heightened in these populations. Among PLWH, sexual minority men and transgender women are disproportionately impacted; sexual minority men comprised 71% of new HIV infections in the USA in 2022 [41] and 36.5% of all new HIV diagnoses in 2021 in the UK [42], and it was estimated that, in 2022, the global risk of acquiring HIV among transgender women was 20 times higher than that among the general adult population [42]. A systematic review and meta-analysis reported that, in the USA, an estimated 44% of Black transgender women have HIV, significantly higher than that of White transgender women (6.7%) and transgender women of other races and ethnicities (9.8%) [43]. This staggering inequity is a concrete example of UNAIDS’ finding that HIV disparities—and thus potential HIV-associated diabetes risk—are worsened among minoritised individuals across the globe [44]. Therefore, an intersectional lens is recommended when considering the link between HIV and metabolic health among SGM individuals.

#### Inflammation and stress

While prior studies have reported that under-represented racial and ethnic groups have higher chronic inflammation levels and greater risks for chronic conditions tied to discrimination [45], there have been limited investigations in SGM populations. A review found that social stigma, such as exclusion, denigration and rejection, towards SGM people can promote systemic inflammation [46]. The few studies that have characterised inflammation in SGM populations have reported high levels of inflammatory markers (e.g. C-reactive protein [CRP], IL-6) [47], attributed to stress and discrimination [47]. High inflammation levels can decrease adiponectin levels and lead to insulin resistance [48], which is associated with increased type 2 diabetes risk. For TGD populations, testosterone may increase levels of proinflammatory cytokines and cardiometabolic risk [49]. As with HIV, future studies should investigate inflammatory markers among SGM populations with intersecting marginalised identities.

### Diabetes in SGM populations

Despite disparities in cardiometabolic risk factors, there is disagreement in published literature about whether SGM populations have a higher prevalence of diabetes than non-SGM counterparts (Table 2); in addition, to our knowledge, no large studies of diabetes in SGM populations have distinguished between type 1 and type 2 diabetes [50–54]. The majority of diabetes prevalence estimates are derived from US data and meta-analyses thereof. However, data from the UK-based GP Patient Survey estimate that, as a group, sexual minority women have 1.2 times the odds of prevalent diabetes compared with heterosexual women [53]; driven by estimates in bisexual and other queer women rather than gay/lesbian women (Table 2), this is consistent with the conceptual model (Fig. 1), which would expect to see higher rates of diabetes among marginalised populations. In contrast, UK-based GP Patient Survey data do not indicate that sexual minority men as a group have an increased prevalence of diabetes compared with heterosexual men, although bisexual and other queer men have an increased diabetes prevalence compared with heterosexual men (Table 2) [53].

**Table 2:** Estimates of prevalent diabetes in SGM populations

Country	First author (year)	Data source	Comparison	OR (95% CI)
UK	Saunders (2021) [54]	2015–2017 GP Patient Survey	SM women vs heterosexual women	1.2 (1.2, 1.3)*
			Gay/lesbian women vs heterosexual women	1.0 (0.9, 1.2)
			Bisexual women vs heterosexual women	1.3 (1.2, 1.5)*
			Other queer women vs heterosexual women	1.3 (1.2, 1.4)*
			SM men vs heterosexual men	1.0 (1.0, 1.1)
			Gay men vs heterosexual men	0.9 (0.9, 1.0)
			Bisexual men vs heterosexual men	1.2 (1.1, 1.3)*
			Other queer men vs heterosexual men	1.1 (1.0, 1.2)*
	Saunders (2023) [53]	2021 GP Patient Survey	TGD vs cisgender individuals	1.2 (1.1, 1.4)*
USA	Haarmann (2023) [51]	Meta-analysis of eight studies^a^	SM women vs heterosexual women	0.8 (0.7, 0.9)*
			Lesbian women vs heterosexual women	0.9 (0.7, 1.2)
			Bisexual women vs heterosexual women	0.7 (0.6, 0.9)*
	Haarmann (2024) [100]	Meta-analysis of ten studies	SM men vs heterosexual men	1.0 (0.8, 1.1)
			Gay men vs heterosexual men	0.9 (0.8, 1.1)
			Bisexual men vs heterosexual men	1.0 (0.7, 1.4)
	Meads (2018) [6]	Meta-analysis of seven studies	Bisexual women vs heterosexual women	0.7 (0.5, 0.9)*
			Lesbian women vs heterosexual women	0.9 (0.7, 1.1)
	Heslin (2021) [30]	2017–2019 BRFSS	SM adults vs heterosexual adults	1.1 (1.0, 1.2)
	Nokoff (2018) [55]	2015 BRFSS	Transgender men vs cisgender women	0.6 (0.3, 1.2)
			Transgender men vs cisgender men	0.4 (0.2, 0.9)*
			Transgender women vs cisgender women	1.7 (0.9, 3.0)
			Transgender women vs cisgender men	1.2 (0.7, 2.2)
			Gender-diverse AMAB vs cisgender men	0.4 (0.1, 1.1)
			Gender-diverse AFAB vs cisgender women	1.1 (0.2, 5.6)
	Tran (2023) [52]	All of Us	SM men vs heterosexual men	0.9 (0.9, 1.0)*
			SM women vs heterosexual women	0.9 (0.8, 0.9)*
			Gender-diverse AFAB vs heterosexual men	0.7 (0.5, 0.9)*
			Gender-diverse AFAB vs heterosexual women	0.6 (0.5, 0.8)*
			Gender-diverse AMAB vs heterosexual men	0.7 (0.5, 1.1)
			Gender-diverse AMAB vs heterosexual women	0.6 (0.4, 1.0)*
			Transgender men vs cisgender men	1.2 (0.9, 1.5)
			Transgender women vs cisgender women	0.9 (0.7, 1.1)

^a^One of the included studies used data from Australia

^*^Indicates a significant association with *p*<0.05

AFAB, assigned female at birth; AMAB, assigned male at birth; SM, sexual minority; TGD, transgender and gender diverse

Much of the US data arise from analyses of BRFSS data, which incorporate survey weights so that results are representative of the US population. Results suggest that, in aggregate, sexual minority adults have a 10% greater odds of diabetes prevalence than heterosexual adults (Table 2) [30], although in race-stratified analyses this association was significant only in non-Hispanic White individuals. Sex-specific analyses suggest the opposite to the findings of the GP Patient Survey such that sexual minority men, but not women, had an increased diabetes prevalence compared with their heterosexual counterparts. Similarly, conflicting results are observed for TGD individuals, with GP Patient Survey data showing that TGD adults had 1.2 times the odds of prevalent diabetes compared with cisgender adults [52], and BRFSS data showing that, compared with cisgender men, transgender women had no difference in diabetes prevalence and transgender men had a decreased diabetes prevalence [55].

Epidemiological studies investigating diabetes using objective measures (e.g. diabetes medication use, HbA_1c_) have found related disparities impacting sexual minority women. In the Nurses’ Health Study II, sexual minority women had a significantly earlier age of diabetes onset than heterosexual peers [56]. The US-based Add Health cohort study found that women who reported same-sex sexual attraction and/or sexual minority sexual identity in both adolescence and adulthood were more likely to have prevalent diabetes than their heterosexual female counterparts [57]. However, studies conducted using electronic health record (EHR) data from the All of Us study in the USA have found a lower diabetes prevalence among SGM than non-SGM participants (Table 2) [52]. The variation in these findings may be due in part to differences in study samples, study designs, data sources (e.g. EHR vs self-report) and methodology and, generally, the use of small sample sizes.

Given that many studies report that SGM individuals are less likely to access healthcare due to lack of health insurance, inability to pay and fear of poor-quality treatment, SGM individuals may be less likely to be aware that they have diabetes [58, 59]. Studies using only self-report data to assess diabetes prevalence (e.g. BRFSS, NHIS) in populations with less access to healthcare are biased towards undercounting diabetes cases and towards null findings [60, 61]. Studies that include both self-report of diabetes and laboratory testing (e.g. fasting plasma blood glucose, HbA_1c_) at the population level may help overcome this weakness. In addition, although EHR data contain objective measures of glycaemic status, only individuals who have gained access to healthcare, disclosed their SGM status to healthcare providers and had their SGM data recorded within their EHR would be classified as SGM in the data. Although studies investigating diabetes in safety-net healthcare systems can help address some of these issues, studies seeking to describe potential diabetes disparities in SGM populations in EHR datasets may be biased toward the null.

Finally, with over 90% of all diabetes cases attributable to type 2 diabetes, and a higher incidence of type 2 diabetes among adults aged 45 and older, most studies investigating diabetes disparities in SGM populations are subject to analytical limitations, as they often do not account for differences in the mean age of SGM compared with non-SGM populations. In nearly all epidemiological surveillance and EHR studies conducted to date, SGM participants have been significantly younger than their non-SGM counterparts [52].

### Outcomes following diabetes

Diabetes is an established risk factor for CVD (i.e. myocardial infarction [MI] and stroke); people with diabetes are twice as likely to have heart disease or a stroke compared with those without the condition. Chronic kidney disease (CKD) is also a common long-term complication of diabetes. The US Centers for Disease Control and Prevention (CDC) estimates that one-third of adults with diabetes have or will develop CKD [62]; Diabetes UK puts this figure at 40% [63]. Below, we present what is currently known about prevalent CVD and CKD in SGM populations; there is a dearth of literature on incident CVD and CKD in SGM individuals.

#### CVD: MI and stroke

Several studies report excess CVD risk in sexual minority adults compared with heterosexual peers, but whether they have a greater prevalence of CVD events is unclear (Table 3). Conflicting results likely arise due to ascertainment of sexual orientation (self-reported or identified via natural language processing) and history of CVD events (self-reported or based on diagnosis codes). Mereish et al found that mastery, one’s sense of control over one’s life, mediated the association between minority stressors and CVD risk in an SGM population, such that increased minority stressors decreased one’s sense of mastery, which was then associated with greater CVD risk [64], a finding that is consistent with our conceptual model (Fig. 1).

**Table 3:** Association of sexual orientation and gender identity with prevalent CVD

First author (year)	Sample	Outcome	Comparison	OR (95% CI)
Meads (2018) [6]	Meta-analysis of five studies	Prevalent CVD	Lesbian women vs heterosexual women	0.94 (0.73, 1.21)
			Bisexual women vs heterosexual women	0.90 (0.54, 1.51)
Caceres (2019) [104]	2014–2016 BRFSS	Prevalent MI	Gay men vs heterosexual men	1.04 (0.78, 1.38)
			Bisexual men vs heterosexual men	1.19 (0.81, 1.75)
			Gay/lesbian women vs heterosexual women	0.62 (0.40, 0.97)*
			Bisexual women vs heterosexual women	1.00 (0.63, 1.58)
		Prevalent stroke	Gay men vs heterosexual men	0.96 (0.65, 1.39)
			Bisexual men vs heterosexual men	1.52 (0.96, 2.41)
			Gay/lesbian women vs heterosexual women	1.17 (0.75, 1.82)
			Bisexual women vs heterosexual women	1.46 (1.01, 2.12)*
Chandra (2023) [72]	2014–2019 BRFSS	Prevalent MI	SM women vs heterosexual women	1.32 (1.10, 1.59)*
			SM men vs heterosexual men	0.95 (0.85, 1.07)
		Prevalent stroke	SM women vs heterosexual women	1.36 (1.16, 1.59)*
			SM men vs heterosexual men	1.17 (1.00, 1.36)*
Streed (2024) [96]	Veterans Healthcare Administration	Prevalent CVD	Sexual minority vs heterosexual adults	1.17 (1.13, 1.21)*
			SM women vs heterosexual women	1.15 (1.00, 1.31)*
			SM men vs heterosexual men	1.17 (1.13, 1.22)*
		Prevalent MI	Sexual minority vs heterosexual adults	1.14 (1.03, 1.27)*
			SM women vs heterosexual women	1.09 (0.67, 1.78)
			SM men vs heterosexual men	1.15 (1.03, 1.27)*
		Prevalent stroke	Sexual minority vs heterosexual adults	1.18 (1.13, 1.22)*
			SM women vs heterosexual women	1.14 (0.99, 1.31)
			SM men vs heterosexual men	1.18 (1.14, 1.23)*
Tran (2023) [52]	All of Us	Prevalent CVD	SM men vs heterosexual men	0.99 (0.93, 1.07)
			SM women vs heterosexual women	0.98 (0.90, 1.07)
			Gender-diverse AFAB vs heterosexual men	0.62 (0.42, 0.91)*
			Gender-diverse AFAB vs heterosexual women	0.99 (0.67, 1.45)
			Gender-diverse AMAB vs heterosexual men	0.83 (0.52, 1.32)
			Gender-diverse AMAB vs heterosexual women	1.32 (0.82, 2.12)
			Transgender men vs cisgender men	0.68 (0.49, 0.94)
			Transgender women vs cisgender women	1.10 (0.83, 1.46)
Caceres (2020) [61]	2014–2017 BRFSS	Prevalent CVD	Transgender women vs cisgender men	1.38 (1.01, 1.88)*
			Transgender men vs cisgender men	0.97 (0.52, 1.81)
			Gender-diverse vs cisgender men	1.11 (0.53, 2.30)
			Transgender women vs cisgender women	2.24 (1.65, 3.06)*
			Transgender men vs cisgender women	1.60 (0.87, 2.93)
			Gender-diverse vs cisgender women	1.67 (0.82, 3.40)
Alzahrani (2019) [69]	2014–2017 BRFSS	Prevalent MI	Transgender women vs cisgender men	1.32 (0.92, 1.90)
			Transgender men vs cisgender men	2.53 (1.14, 5.63)*
			Transgender women vs cisgender women	2.56 (1.78, 3.68)*
			Transgender men vs cisgender men	4.90 (2.21, 10.90)*
Streed (2025) [70]	Veterans Healthcare Administration	Incident CVD	Transgender adults vs cisgender women	1.25 (1.19, 1.32)*^a^
			Transgender adults vs cisgender men	0.96 (0.92, 0.99)*^a^
			Transmasculine adults vs cisgender women	1.00 (1.00, 1.22)^a^
			Transmasculine adults vs cisgender men	0.86 (0.79, 0.94)*^a^
			Transfeminine adults vs cisgender women	1.28 (1.16, 1.42)*^a^
			Transfeminine adults vs cisgender men	0.91 (0.83, 1.00)^a^
van Zijverden (2024) [71]	Meta-analysis of 22 studies	Incident CVD	Transgender women vs cisgender men	1.4 (1.0, 1.9)*^b^
			Transgender men vs cisgender women	1.4 (1.1, 1.8)*^b^
		Incident stroke	Transgender women vs cisgender men	1.3 (1.0, 1.8)^b^
			Transgender men vs cisgender women	1.3 (1.0, 1.6)^b^
		Incident MI	Transgender women vs cisgender men	1.0 (0.8, 1.2)^b^
			Transgender men vs cisgender women	1.7 (0.8, 3.6)^b^
		Thrombosis	Transgender women vs cisgender men	2.2 (1.1, 4.5)*^b^
			Transgender men vs cisgender women	1.4 (1.0, 2.0)*^b^

^a^HR reported rather than OR

^b^RR reported rather than OR

^*^Indicates a significant association with *p*<0.05

AFAB, assigned female at birth; AMAB, assigned male at birth; SM, sexual minority

The prevalence and incidence of CVD among TGD adults has been investigated more than CVD among sexual minority adults. While early studies found no association between GAHT and MI incidence and mortality risk [65, 66], subsequent studies consistently demonstrated an increased prevalence of CVD among transgender women receiving GAHT [67]; such results were likely due to use of ethinyloestradiol as GAHT among transgender women [68]. Since ethinyloestradiol has been replaced by lower dose transdermal or oral bioidentical oestrogen, an increased prevalence of CVD among transgender women receiving GAHT has not been demonstrated compared with transgender women not receiving GAHT [68]. Regardless of GAHT use, data from the BRFSS consistently demonstrate that transgender women have an increased prevalence of CHD, stroke and MI compared with cisgender women and an increased prevalence of any CVD compared with cisgender men [61, 69]; gender non-conforming adults have increased odds of MI vs cisgender women [61]; and transgender men have 2.5 and 4.9 times the odds of MI compared with cisgender men and cisgender women, respectively [69]. Data from the US Veterans Healthcare Administration suggest that TGD veterans’ CVD risk is greater than that of cisgender women but lower than that of cisgender men and that, among TGD veterans, receipt of masculinising GAHT is associated with decreased CVD risk but feminising GAHT is not associated with CVD risk [70]. A 2024 meta-analysis of 22 studies reported that the overall incidence of CVD (stroke, MI or thrombosis) was greater in transgender women than cisgender men and in transgender men than cisgender women. However, these associations were driven largely by the associations between gender identity and thrombosis, as the 95% CIs for MI and stroke included the null value of 1.0 [71].

#### Chronic kidney disease

Few studies have investigated CKD disparities impacting SGM populations (Table 4) but most report an increased prevalence of CKD in SGM individuals compared with similar heterosexual cisgender individuals. Results from All of Us are a notable exception; in this study, most associations were null or suggested that sexual minority women and gender-diverse individuals assigned male at birth were at lower risk of CKD than heterosexual women and men, respectively (Table 4) [52]. However, these results may be driven by the significantly younger mean age of sexual minority women and gender-diverse people compared with the mean ages of other groups [52]. Analysis of 2014–2019 BRFSS data showed that older sexual minority men were more likely to report CKD than similarly aged heterosexual men; this disparity was attenuated after adjustment for diabetes status [72], suggesting that CKD disparities may be rooted in a higher prevalence of diabetes in SGM individuals. In a cross-sectional study of TGD individuals, the estimated prevalence of CKD was found to be 36% [73], more than twice the CKD prevalence (15%) in the general US and UK populations. Interestingly, the prevalence of CKD was greater in transgender men and women who were not receiving GAHT than in those who were. These results are in contrast to studies which observed that creatinine significantly decreased over the first year of GAHT usage among transgender women, yet significantly increased over the same time period in transgender men [74, 75]. Because GAHT may impact creatinine, GFR should be measured directly rather than estimated to accurately guide clinical decision-making among TGD individuals [67].

**Table 4:** Association of sexual orientation and gender identity with prevalent CKD

Country	First author (year)	Sample	Comparison	OR (95% CI)
UK	Saunders (2021) [54]	2015–2017 GP Patient Survey	SM women vs heterosexual women	1.4 (1.2, 1.5)*
			SM men vs heterosexual men	1.4 (1.3, 1.5)*
			Gay men vs heterosexual men	1.4 (1.2, 1.5)*
			Bisexual men vs heterosexual men	1.7 (1.4, 2.0)*
			Other queer men vs heterosexual men	1.2 (1.0, 1.4)
	Saunders (2023) [53]	2021 GP Patient Survey	TGD vs cisgender adults	1.4 (1.2, 1.6)*
USA	Heslin (2021) [30]	2017–2019 BRFSS	SM adults vs heterosexual adults	1.47 (1.25, 1.69)*
	Chandra (2023) [72]	2014–2019 BRFSS	SM women vs heterosexual women	1.16 (0.98, 1.37)
			SM men vs heterosexual men	1.30 (1.09, 1.54)*
	Tran (2023) [52]	All of Us	SM men vs heterosexual men	1.06 (0.97, 1.16)
			SM women vs heterosexual women	0.83 (0.73, 0.93)*
			Gender-diverse AFAB vs heterosexual men	0.63 (0.39, 1.02)
			Gender-diverse AFAB vs heterosexual women	0.92 (0.58, 1.48)
			Gender-diverse AMAB vs heterosexual men	0.37 (0.15, 0.89)*
			Gender-diverse AMAB vs heterosexual women	0.54 (0.22, 1.30)
			Transgender men vs cisgender men	0.87 (0.61, 1.25)
			Transgender women vs cisgender women	1.11 (0.79, 1.57)

^*^Indicates a significant association with *p*<0.05

AFAB, assigned female at birth; AMAB, assigned male at birth; SM, sexual minority; TGD, transgender and gender diverse

### Diabetes complications and quality of care

Diabetes leads to severe complications that affect quality of life, particularly because of the impact on vasculature and functional and cognitive disability. Fortunately, these complications can be delayed or entirely prevented with good glycaemic and comorbidity management. Successful management of diabetes requires improvement in self-management skills, retention in healthcare, access to pharmacological interventions, and addressing psychosocial issues. However, SGM individuals face several barriers that affect successful diabetes management.

Structural factors hinder SGM individuals from accessing or engaging with care. SGM individuals experience higher rates of poverty than non-SGM populations [76], and frequently face policies that discriminate against them [77], affecting healthcare access. In the USA, SGM adults living in jurisdictions without non-discrimination laws have lower health insurance coverage than their non-SGM counterparts [78]. Policies can limit the ability of SGM individuals to share insurance as well as involve SGM partners in their care [79]. The latter is especially relevant when considering that people with diabetes are at risk of rapid-onset life-threatening emergencies where cognition is severely affected or in settings of end-stage disease that require management within intensive nursing or palliative care facilities. Policies also create stigmatising environments associated with increased risk of victimisation [80] and adverse health outcomes [81]. These issues are further complicated by immigration policies that limit access to insurance and care. Finally, many areas criminalise SGM identities, leading to direct harm and stress. These laws also force people not to reveal their sexual orientation and to delay or forego healthcare [82].

In addition to these structural issues, SGM individuals experience interpersonal discrimination in healthcare interactions [83], including healthcare encounters in which they are assumed to be straight and/or cisgender [84]. To maintain their safety, SGM individuals sometimes need to conceal their identity, which disrupts the patient–physician relationship [83]. As a result, SGM people avoid seeking care due to anticipated or experienced discrimination in healthcare [85], resulting in many not receiving essential healthcare [86], including screening for diabetes [87].

In addition to the high likelihood that SGM people with diabetes have issues receiving healthcare [88], an analysis of EHR data showed that transgender men and women with diabetes were less likely to receive HbA_1c_ re-testing than cisgender men with diabetes [88]. It was also reported that sexual minority women with diabetes were less likely to receive HbA_1c_ re-testing than gay men. Further, both sexual minority women and heterosexual men were less likely to achieve HbA_1c_ <75 mmol/mol (<9%) than gay men [88]. A common theme in the research on quality of diabetes care is the heterogeneity of outcomes by sexual orientation and gender identity, highlighting the importance of stratifying results when possible. There are very few publications on risk of diabetes complications outside studies on CKD and CVD.

### Interventions

Interventions for diabetes rely on providing education and coaching on lowering BMI and achieving glycaemic control through self-management practices. However, self-management techniques for diabetes can be difficult to execute, particularly without social support [89]. For many, including SGM individuals, successful health outcomes require social support [89]. In the USA, the National LGBT Health Education Center recommends community support through social interaction for diabetes management and prevention [90], such as support groups where SGM participants can exchange information on providers and encourage healthy practices, such as walking or running groups.

To our knowledge, there are few diabetes management or prevention efforts centred on SGM people. Existing diabetes interventions for SGM individuals should be multilevel, similar to smoking cessation programmes for SGM people [91]. Community-centred elements that improve intervention success include using SGM community members for intervention implementation and providing space for discussing unique factors affecting behaviour. Furthermore, interventions that account for multiple risk factors may be able to address several risks simultaneously through integrated strategies. Taking a gender-inclusive vs gender-specific approach to understanding shared vs unique risk factors, respectively, per gender group is a necessary part of developing interventions in this area. Risk reduction programmes for TGD populations, for example, that address HIV risk and substance use while also targeting transphobic structural and social factors unique to this population and its subgroups can be effective at providing education and promoting healthy behaviours.

## Discussion

### Summary

This narrative review reveals significant variation in cardiometabolic risk and outcomes among SGM subpopulations. Studies of UK data find that sexual minority women have an increased risk of diabetes compared with heterosexual women, while data from the USA reveal ambiguous results based on data source. Transgender adults were found to be at increased risk for diabetes compared with cisgender comparators in UK data sources but not in the USA. For CKD, risk varied by subpopulation and data source. Notably, SGM subpopulations in the UK were reported to have an increased risk of CKD while similar populations in the USA appear to have no difference in risk compared with cisgender, heterosexual comparators. Similarly, risk for CVD among sexual minority populations varied by data source. However, both US and UK data suggest that transgender adults are at increased risk of CVD.

### Practical and methodological limitations of evidence to date

Although we included data from the UK where possible, most of the data describing the cardiometabolic health of SGM populations comes from US-based samples; data from countries outside the UK and USA are even more limited. This lack of UK data combined with cultural and structural differences between the UK and the USA, largely arising from differences in healthcare systems and access, limit the generalisability of these findings to UK individuals and make it hard to compare findings across countries. Further, given the recent political shift in the USA, particularly Executive Order 14168 and the cancellation of federally funded grants focused on SGM health, the future of research on this topic in the USA is uncertain [92, 93]. It is imperative that non-US researchers aid in moving this research forward.

In addition to the practical challenges researchers face when studying SGM health, there are also methodological limitations to consider. Most SGM research relies on cross-sectional data analysis and assessment of disease prevalence rather than longitudinal data analysis, which would enable the assessment of disease incidence. This lack of data on incident diabetes and development of CVD and CKD following a diabetes diagnosis prevents us from drawing conclusions surrounding temporality. The findings of this review highlight the need for longitudinal cohort data in which sexual orientation and gender identity are self-reported and cardiometabolic diagnoses are objectively ascertained through chart review to close these gaps in knowledge and aid in the identification of intervention points to lessen disparities in SGM health. However, the proportion of SGM individuals in cohort studies is typically low and often too small to ensure adequate statistical power to detect true significant differences. In the absence of cohort data, longitudinal analysis of EHRs with cardiometabolic outcomes ascertained via diagnosis codes may show promise.

However, while EHRs resolve sample size concerns, they have their own limitations. Individuals identified as SGM in EHRs are younger, on average, than individuals identified as heterosexual cisgender [70, 94–97]. Failure to adjust for age then leads to biased results, and even covariate adjustment for age may be inadequate to fully account for age differences between groups if the age distribution is sufficiently disparate between groups. Thus, creation of a nested age-matched sample may be necessary to eliminate the confounding effect of age. Further, EHRs typically contain little to no information on social determinants of health and, as SGM populations are disproportionately affected by lower socioeconomic status, income and healthcare access [98, 99], inadequate adjustment for these confounders may obscure the specific impact of sexual orientation and gender identity on outcomes.

Finally, although we reviewed and included data from meta-analyses, it is important to note that some of the estimates included in the meta-analyses were based on comparisons where the SGM group under study had fewer than ten events, which may have biased the results [51, 100]. Meta-regressions that adjust for study characteristics may help reduce some of this bias.

### Future directions and conclusion

There is an urgent need for research into sexual orientation, gender identity, and cardiometabolic risk and, subsequently, tailored interventions that consider the intersecting social locations and systems of oppression that shape the challenges and needs of SGM communities. Future research must include SGM populations in the design while also including sexual orientation and gender identity data in the routine collection of demographic characteristics of study samples. Additionally, research that considers both general and SGM-specific vulnerabilities and identifies health-promoting resiliencies should inform future intervention targets, which must be adapted to the needs of SGM subpopulations. Finally, SGM health content should be included in the training, accreditation and qualification requirements for health professions [101, 102]. There are exciting opportunities for future research and clinical and public health efforts to better understand and reduce cardiometabolic disparities among SGM people.

## Supplementary Information

Below is the link to the electronic supplementary material.

ESM Table (PDF 94 KB)

Figure slide (PPTX 216 KB)

## Abbreviations

Add Health: National Longitudinal Study of Adolescent Health
ART: Antiretroviral therapy
CKD: Chronic kidney disease
EHR: Electronic health record
GAHT: Gender-affirming hormone therapy
LGB: Lesbian, gay and bisexual
LGBTQ+: Lesbian, gay, bisexual, transgender or queer
MI: Myocardial infarction
NHANES: National Health and Nutrition Examination Survey
NHIS: National Health Interview Survey
PLWH: People living with HIV
SGM: Sexual and gender minority
TGD: Transgender and gender diverse

## Authors’ relationships and activities

CS reports receiving consulting fees from Everlywell, L’Oreal, the Texas Health Institute, the Research Institute for Gender Therapeutics and the US Department of Justice unrelated to this work. HLW is serving in their personal capacity. The other authors declare that there are no relationships or activities that might bias, or be perceived to bias, their work. The views expressed in this article are those of the authors and do not necessarily reflect the position or policies of the funders or institutions or any agencies of the US Government.
