# Supplementary material for: Sexual orientation, gender identity and cardiometabolic risk: a narrative review
Source: Diabetologia. 2025 Oct 21;68(12):2652–69. doi: 10.1007/s00125-025-06572-7 (PMC12594680; doi:10.1007/s00125-025-06572-7)
Supplement: Supplementary file 1 — ESM Table (PDF 94 KB) [file 125_2025_6572_MOESM1_ESM.pdf]

ESM Table 1. Search Strategies

**Section**	**Databases**	**Years**	**Search Terms**
Risk Factors for Diabetes	PubMed	2017-2024	(("Smoking"[Mesh] or "Smoking"[Tiab] or "HIV"[Mesh] or "HIV"[Tiab] or "Diet"[Mesh] or "Diet"[Tiab] or "Exercise"[Mesh] or "Exercise"[Tiab] or "Leisure Activities"[Mesh] or "Leisure Activities"[Tiab] or "Body Mass Index"[Mesh] or "Body Mass Index"[Tiab] or "Adiposity"[Mesh] or "Adiposity"[Tiab] or "Sleep"[Mesh] or "Sleep"[Tiab])) AND (("Sexual and Gender Minorities"[Mesh] or "Sexual and Gender Minorities"[Tiab] or "Bisexuality"[Mesh] or "Homosexuality"[Mesh] or "Transgender Persons"[Mesh] or "Transsexualism"[Mesh] or "LGBT*"[Tiab] or "Gay" [Mesh] or "Lesbian" [Mesh] or "Bisexual" [Mesh] or "Sexual Orientation" [Mesh] or "Gender Identity" [Mesh]))
Outcomes Following Diabetes	PubMed	2017-2024	(("Sexual and Gender Minorities"[Mesh] or "Sexual and Gender Minorities"[Tiab] or "Bisexuality"[Mesh] or "Homosexuality"[Mesh] or "Transgender Persons"[Mesh] or "Transsexualism"[Mesh] or "LGBT*"[Tiab] or "Gay" [Mesh] or "Lesbian" [Mesh] or "Bisexual" [Mesh] or "Sexual Orientation" [Mesh] or "Gender Identity" [Mesh])) AND ("Myocardial Infarction"[Mesh] or "Myocardial Infarction"[Tiab] or "Heart Attack" [Mesh] or "Heart Attack" [Tiab] or "Stroke"[Mesh] or "Stroke"[Tiab] or "Kidney Disease"[Mesh] or "Kidney Disease"[Tiab])
Diabetes complications and quality of care	PubMed	No restriction	("lesbian" OR "gay" OR "bisexual" OR "transgender" OR "queer" OR "sexual minorit*" OR "gender minorit*" OR "sexual and gender minorit*") AND (diabetes, diabet*, chronic kidney disease, quality of care, complication, sequelae)
