# Supplementary material for: Sexual orientation, gender identity and cardiometabolic risk: a narrative review
Source: Diabetologia. 2025 Oct 21;68(12):2652–69. doi: 10.1007/s00125-025-06572-7 (PMC12594680; doi:10.1007/s00125-025-06572-7)
Supplement: Supplementary file 2 — Figure slide (PPTX 216 KB) [file 125_2025_6572_MOESM2_ESM.pptx]

## Slide 1
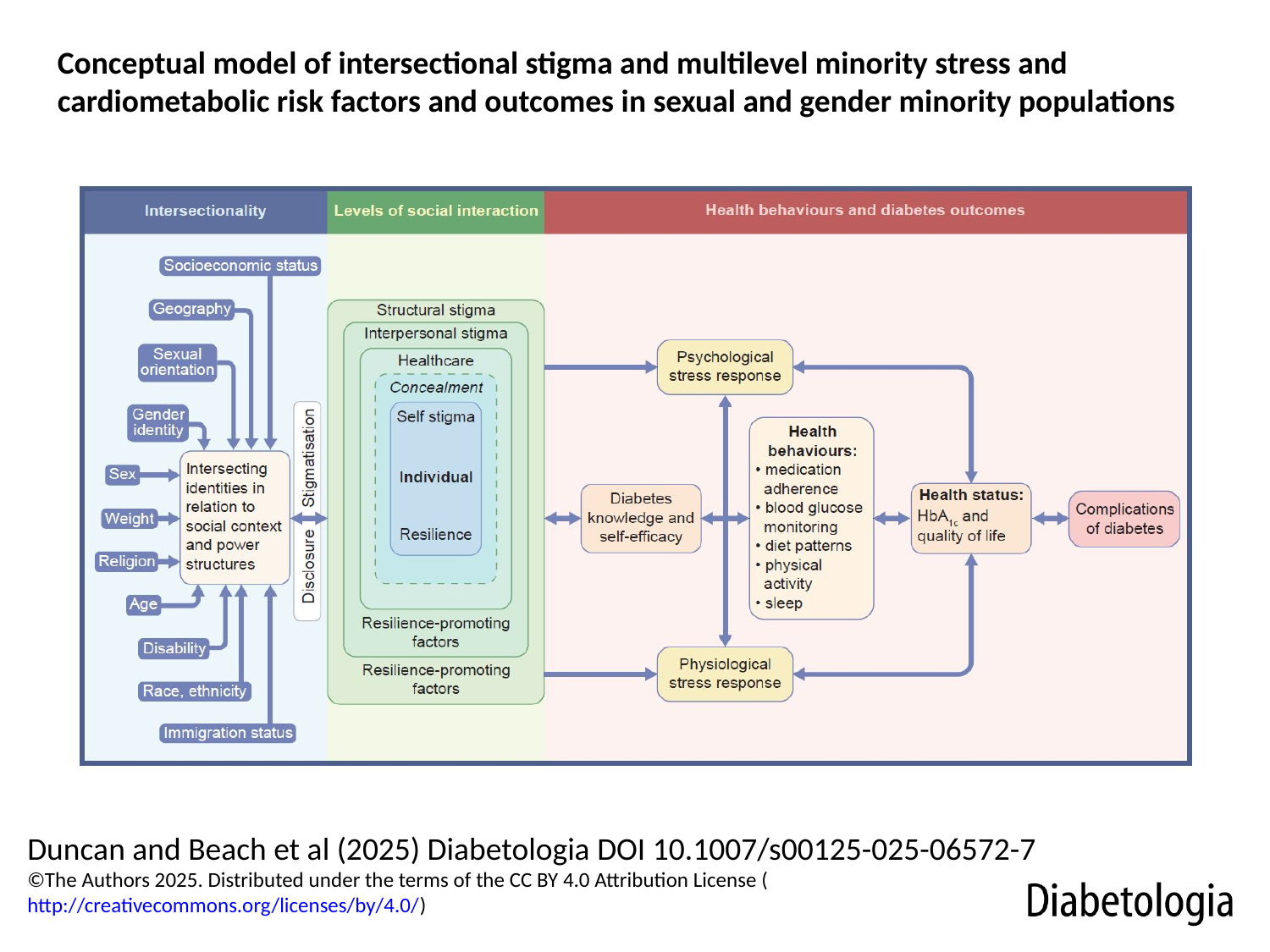

Conceptual model of intersectional stigma and multilevel minority stress and cardiometabolic risk factors and outcomes in sexual and gender minority populations
Duncan and Beach et al (2025) Diabetologia DOI 10.1007/s00125-025-06572-7
©The Authors 2025. Distributed under the terms of the CC BY 4.0 Attribution License (http://creativecommons.org/licenses/by/4.0/)
